# Seizure outcome in temporal glioblastoma surgery: lobectomy as a supratotal resection regime outclasses conventional gross-total resection

**DOI:** 10.1007/s11060-021-03705-x

**Published:** 2021-02-07

**Authors:** Valeri Borger, Motaz Hamed, Inja Ilic, Anna-Laura Potthoff, Attila Racz, Niklas Schäfer, Erdem Güresir, Rainer Surges, Ulrich Herrlinger, Hartmut Vatter, Matthias Schneider, Patrick Schuss

**Affiliations:** 1 Department of Neurosurgery, University Hospital Bonn, Venusberg-Campus 1, 53127 Bonn, Germany; 2 Department of Epileptology, University Hospital Bonn, Bonn, Germany; 3 Division of Clinical Oncology, Department of Neurology, University Hospital Bonn, Bonn, Germany

**Keywords:** Glioblastoma, Seizure outcome, Supra-marginal resection

## Abstract

**Introduction:**

The postoperative seizure freedom represents an important secondary outcome measure in glioblastoma surgery. Recently, supra-total glioblastoma resection in terms of anterior temporal lobectomy (ATL) has gained growing attention with regard to superior long-term disease control for temporal-located glioblastoma compared to conventional gross-total resections (GTR). However, the impact of ATL on seizure outcome in these patients is unknown. We therefore analyzed ATL and GTR as differing extents of resection in regard of postoperative seizure control in patients with temporal glioblastoma and preoperative symptomatic seizures.

**Methods:**

Between 2012 and 2018, 33 patients with preoperative seizures underwent GTR or ATL for temporal glioblastoma at the authors’ institution. Seizure outcome was assessed postoperatively and 6 months after tumor resection according to the International League Against Epilepsy (ILAE) classification and stratified into favorable (ILAE class 1) versus unfavorable (ILAE class 2–6).

**Results:**

Overall, 23 out of 33 patients (70%) with preoperative seizures achieved favorable seizure outcome following resection of temporal located glioblastoma. For the ATL group, postoperative seizure freedom was present in 13 out of 13 patients (100%). In comparison, respective rates for the GTR group were 10 out of 20 patients (50%) (p = 0.002; OR 27; 95% CI 1.4–515.9).

**Conclusions:**

ATL in terms of a supra-total resection strategy was associated with superior favorable seizure outcome following temporal glioblastoma resection compared to GTR. Regarding above mentioned survival benefit following ATL compared to GTR, ATL as an aggressive supra-total resection regime might constitute the surgical modality of choice for temporal-located glioblastoma.

## Introduction

Seizures are the most common symptoms in patients with malignant brain tumors and often constitute the first clinical manifestation of an intracranial lesion. Tumor location and histopathological classification are decisive for the frequency of seizures. Supratentorial tumors, especially those with superficial and/or temporal localization, are more epileptogenic. However, the incidence of seizures inversely correlate with the degree of malignancy with seizures occurring more frequently in low-grade tumors.

Nevertheless, as neurosurgical, radiological, chemotherapeutic and supportive therapies for glioblastoma have been improved over the last decades [1–5] and overall survival is prolonged, appropriate symptom management is becoming increasingly important in this patient population to avoid negative consequences for the patients' quality of life.

Previous study results indicated that a larger extent of resection might improve the survival of glioblastoma patients [6–8]. In the case of temporally located glioblastomas, it was previously demonstrated that such supra-marginal resection—here in the sense of an anterior temporal lobectomy (ATL) as an epilepsy-surgical therapeutic approach—improves both progression-free and overall survival [9]. Due to the scarcity of data, it remains uncertain to what extent this therapeutic approach has an impact on tumor-related epilepsy (TRE).

Here, we present for the first time data on the influence of this aggressive onco-surgical therapy approach on the outcome of seizures after surgical treatment of temporally located glioblastomas.

## Methods

### Patients

Between 2012 and 2018, all patients with temporal glioblastoma and preoperative seizures aged 18 years or older were included in further analysis. All patients who underwent only biopsy were excluded. After approval by the institutional ethics committee, the medical records were retrospectively reviewed and relevant clinical information were collected and entered into a computerized database (SPSS Version 25, IBM Corp.). These information included patient age, gender, Karnofsky Performance Score (KPS), tumor size and extent, seizure status and semiology (focal vs. generalized) according to the International League Against Epilepsy (ILAE), molecular histopathological parameters and postoperative seizure outcome.

Using the KPS, patients were categorized according to their neurological functional status both preoperatively and in the further postoperative course. KPS ≥ 70 was defined as a favourable outcome during postoperative follow-up immediately after surgery and 3 and 12 months postoperatively.

### Treatment decision

The institutional interdisciplinary central nervous system (CNS) tumor board made the decision on treatment modality on a case-by-case basis at the initial presentation of the patient and during postoperative follow-up. Within the framework of the weekly CNS tumor board, a consensus treatment algorithm is determined for each patient by specialists in neuro-oncology from the fields of neurology, neurosurgery, neuroradiology, neuropathology and radio-oncology. The specification of neurosurgical procedures is primarily based on the individual assessment of the neurosurgeons participating in the CNS tumor board meeting. The decision whether the patient was assigned to ATL or conventional tumor resection was primarily based upon the prevalence of the responsible treating neurosurgeon. Given by the fact, that authors’ institution have a long tradition of epilepsy surgery, those neurosurgeons with epilepsy surgery skills performed more likely ATL. All leading neurosurgeons involved in the surgical treatment of neuro-oncological patients fulfilled the requirements for Neuro-Oncology Centers certificated by the German Cancer Society. Patients with temporal glioblastoma were only selected in this series if the temporal tumor manifestation was within the margin of 4–5 cm in the dominant and 5–6 cm in the non-dominant hemisphere from the temporal pole. Patients with additional infiltration of the mesial temporal lobe structures (amygdala, hippocampus, parahippocampal gyrus and / or entorhinal cortex) in preoperative gadolinium enhanced MR-imaging were also included in the current series. As known from reports on patients with low-grade glioma, the resection of mesial temporal lobe structures is more likely to achieve a better post-operative seizure control [10]. Thus, patients with temporal glioblastoma and evidence of contrast enhancement in mesial temporal lobe on pre-operative MRI underwent additional amygdalohippocampectomy. In patients who had a FLAIR component without any contrast enhancement in the temporo-mesial structures, the removal of these structures was at the discretion of the surgeon and was usually not carried out for epileptological considerations, but rather for neuro-oncological considerations regarding a reduction of the local mass in view of an imminent further therapy such as radiation with the potential danger of subsequent reactive tissue swelling.

### Surgical procedures

For further analysis the patients were divided into two groups: (1) patients undergoing gross-total resection (GTR) of the temporal contrast-enhancing tumor, and (2) those patients who underwent temporal tumor removal by means of additional anterior temporal lobectomy (ATL). ATL was performed with the inclusion of the contrast-enhanced tumor portion as well as unaffected brain parenchyma as indicated by the preoperatively performed magnetic resonance imaging (MRI). Furthermore, in selected cases resection of the temporal glioblastoma with additional amygdalohippocampectomy was performed. In these cases, however, an oncosurgical rather than an epilepsy-surgical therapeutic approach was pursued, which was derived from the accompanying tumor infiltration of the temporo-mesial structures. All surgical procedures were performed using intraoperative neuronavigation, 5-aminolevulinic acid (5-ALA) fluorescence, and if indicated intraoperative neurophysiological monitoring with motor evoked and somatosensory evoked potentials. When performing ATL, the temporal lobe was removed until the margin of maximum 4.5 cm from temporal pole in dominant and 6.5 cm in non-dominant hemisphere [11]. The resection of uncus and amygdala was performed using ultrasound suction system (CUSA) or Penfield dissector, until the pial and arachnoid membranes, adjacent to the crural and ambient cisterns, was reached. The temporal horn was opened using a Penfield dissector followed by entering the ventricle and resection of the anterior part of the hippocampus. Then the en bloc resection of the hippocampus body was performed with the posterior margin at the level of the tectum. The conventional GTR was performed by surgical removing of gadolinium enhanced tumor tissue guided by 5-ALA fluorescence.

### Postoperative care

The extent of resection was evaluated according to the early post-operative MRI within 72 h after surgery. The GTR was defined as complete resection of gadolinium enhancing tissue. In addition, the result of the neuroradiological report of the post-operative MRI examination was taken into account. For this purpose, two independent neuroradiologists, without any knowledge of the clinical course, assessed the neuroimaging. The required information was available for all included patients, extracted from these reports, and categorized according to the above definition. Only patients with GTR were included in the further analysis.

Peri- and postoperative complications were defined as any unfavorable event that occurred within 30 days following initial temporal glioblastoma resection [12].

After histopathological confirmation of glioblastoma, the methylation status of the MGMT promoter was analyzed by pyrosequencing and combined bisulfite restriction analysis.

After surgical removal of the tumor, all patients received adjuvant treatment consisting of radiotherapy, chemotherapy or combined radiochemotherapy, as recommended by the institutional interdisciplinary tumor board.

### Tumor-related seizures

According to ILAE, tumor-associated epilepsy was defined as (1) at least two unprovoked seizures occurring at intervals of > 24 h or (2) one unprovoked seizure with an increased probability of further seizures similar to the general risk after two unprovoked seizures. Therefore, glioblastoma patients with both 1 and > 1 symptomatic seizure were included for further analysis. According to the semiology, the seizures were distinguished into focal aware seizures, focal impaired awareness seizures and focal to bilateral tonic–clonic seizures.

**Fig. 1:**
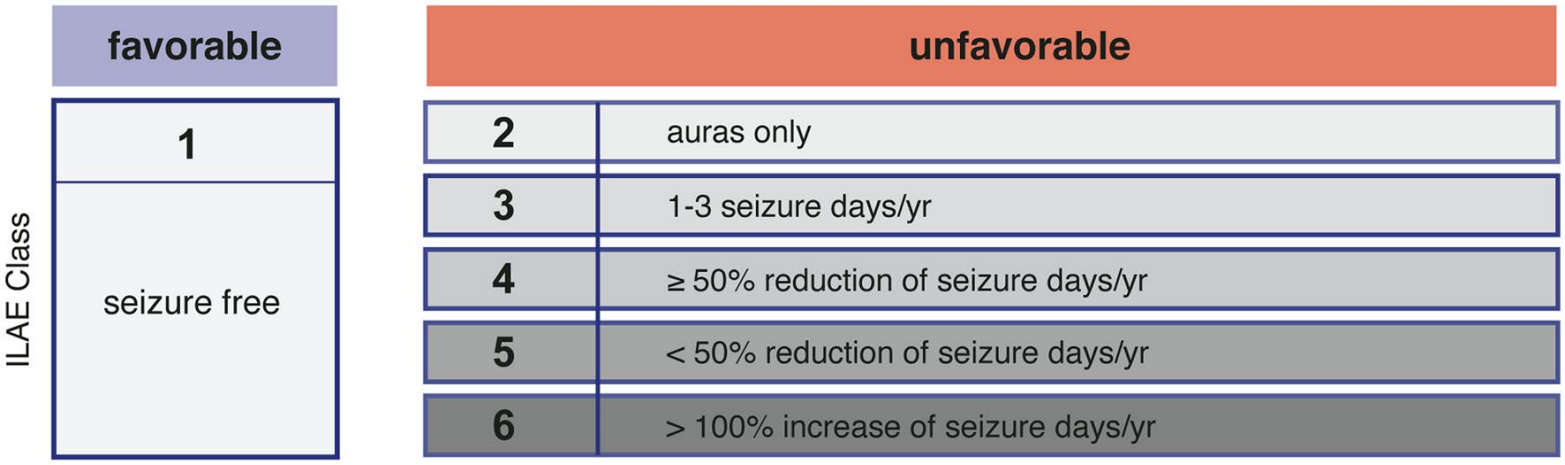
Schematic illustration of the ILAE classification system. Scheme modified from Schneider et al. [31]. ILAE international league against epilepsy

Full seizure control as defined by ILAE Class 1 was determined after 6 months of follow-up instead of the recommended 12 months in the investigated glioblastoma patients and was considered favorable. An unfavorable outcome was defined as ILAE classes 2–6, i.e. pure auras, rare to no improvement and worsening of seizure frequency and/or seizure disabling quality. Detailed information on the ILAE classification system can be found in Fig. 1.

### Statistics

Data analysis was performed using the SPSS (Version 25, IBM Corp.) computer software package. An unpaired t-test was used for parametric statistics. Categorical variables were analyzed in contingency tables using the exact Fisher test. Results with p values < 0.05 were considered statistically significant.

## Results

### Patient characteristics

Between 2012 and 2018, a total of 33 patients with temporally located glioblastoma and preoperative seizures were treated at the authors’ institution. The patients ‘ mean age was 59 ± 14 years. The median KPS score at presentation was 90 (range 60–100). Anterior temporal lobectomy (ATL) was performed in 13 patients (39%), whereas the temporal GTR was performed in 20 patients (61%). The median overall survival (OS) was 22 months (95% CI 11.3–32.7). Further details on patient and tumor characteristics are given in Table 1.

**Table 1:** Patient characteristics

No. of patients	33
Sex	
Male	19 (58)
Female	14 (42)
Mean age (± SD) (in yrs)	59 ± 14
Tumor location	
Dominant hemisphere	19 (58)
Temporomesial infiltration ( +)	17 (52)
Semiology	
Focal aware seizures	12 (36)
Focal impaired-awareness seizures	3 (9)
Focal to bilateral tonic–clonic seizures	18 (55)
Preoperative KPS	
≥ 70	32 (97)
< 70	1 (3)
Surgical modality	
ATL	13 (39)
GTR	20 (61)
MGMT promotor methylation status	
Methylated	16 (48)
Unmethylated	17 (52)
Favorable seizure outcome (ILAE 1)	23 (70)

*ATL* anterior temporal lobectomy, *GTR* gross total resection, *ILAE* International League Against Epilepsy, *KPS* Karnofsky Performance Score, *yrs* years

Values represent number of patients unless otherwise indicated (%)

Surgery-related unfavorable events were present in 4 out of 33 patients (12%). Thereby, postoperative hemorrhage was found in 2 patients (6%) and postoperative meningitis and wound dehiscence in 1 patient, respectively (3%). Among the patients with postoperative hemorrhage, revision surgery was required in one case. Analysis of peri- and postoperative complications did not reveal any profound new language deficit.

### Seizure semiology

As shown in Table 1, the preoperative evaluation revealed focal to bilateral tonic–clonic seizures as a predominantly seizure semiology (18 out of 33 patients (55%)). Most frequently observed were focal aware seizures in 12 out of 33 patients (36%) followed by focal impaired-awareness seizures (3 out of 33 patients (9%)). At admission, all the glioblastoma patients in this series were under antiepileptic drug medication, whereby Levetiracetam was present in 31 patients (94%) and Carbamazepine in 2 patients (6%).

### Seizure outcome

Overall, favorable postoperative seizure outcome in terms of ILAE class 1 was achieved in 23 patients (70%) with temporal glioblastoma and preoperative TRE. There was no statistically significant difference between seizure free patients and patients with continuous seizures according to the preoperative seizure semiology. The analysis of the correlation between seizure control and overall survival (OS) revealed a median OS of 23 months (IQR 13–33.5) in seizure free patients (ILAE 1) vs. 26 months (IQR 3–33.5) in patients with postoperatively persistent seizures (p = 0.34). From the total of 33 patients, adjuvant treatment according to the Stupp protocol was performed in 26 patients (80%), 4 patients (12%) received adjuvant treatment according to the protocol of the CeTeG-trial [13], and 3 patients (9%) received individual adjuvant treatment according to the recommendation of the institutional interdisciplinary tumor board. The comparison of the both outcome groups did not reveal a statistically significant difference. The IDH1 status was available in 22 of 33 patients (66.7%) in this series. From the patients with known IDH1 mutation status, 21 patients (95.5%) were IDH1-wildtype and only 1 patient (4.5%) has an IDH1 mutation. This particulary patient underwent GTR and had persisting seizures (ILAE 2–5) after the surgery. From the total of 21 patients with IDH1-wildtype status, in 9 patients (43%) ATL was performed with a favorable seizure outcome in 15 patients (71.4%). Further details are given in Table 2.

**Table 2:** Seizure outcome

	Favorable seizure outcome (ILAE 1)	Unfavorable seizure outcome (ILAE 2–6)	p value
No. of patients	23 (70)	10 (30)	
Mean age (± SD) (in years)	59 ± 14	60 ± 16	0.96
Female sex	11 (48)	3 (30)	0.46
Tumor location			
Dominant hemisphere	14 (61)	5 (50)	0.71
Temporomesial infiltration ( +)	12 (52)	5 (50)	1.0
Preoperative KPS ≥ 70	22 (96)	10 (100)	1.0
Semiology			0.49
Focal aware seizures	8 (35)	4 (40)	
Focal impaired-awareness seizures	3 (13)	0 (0)	
Focal to bilateral tonic–clonic seizures	12 (52)	6 (60)	
Surgical modality			0.002
ATL	13 (56)	0 (0)	
GTR	10 (44)	10 (100)	
Additonal amygdalo-hippocampectomy	12 (52)	1 (10)	0.05
Unmethylated MGMT promotor status	16 (70)	6 (60)	0.69
Postoperative adjuvant treatment			
Stupp	19 (83)	7 (70)	0.64
CeTeG	3 (13)	1 (10)	1.0
Individual	1 (4)	2 (20)	0.21
Mean hospital stay (± SD) (ds)	16 ± 10	11 ± 7	0.13
mOS (IQR)	22 (13–34)	26 (3–34)	0.34

*ATL* anterior temporal lobectomy, *ds* days*, GTR* gross total resection, *ILAE* International League Against Epilepsy*, IQR interquartile range, KPS* Karnofsky Performance Scale*, mOS* median overall survival, *yrs* years

Values are presented as the number of patients (%) unless stated otherwise

### Influence of additional amygdalohippocampectomy on seizure outcome

A total of 17 patients (52%) suffering from temporally located glioblastoma and preoperative TRE had additional temporo-mesial tumorous infiltration. In detail, 3 of the 17 patients exhibited a contrast-absorbing infiltration of the temporo-mesial structures, whereas 14 of the 17 patients had a FLAIR-correlate in the area of the temporo-mesial structures. In selected patients, additional amygdalohippocampectomy was performed in order to achieve GTR, better relief of space-occupying effect and/or a more favorable seizure outcome. Therefore, 12 of 13 patients (92%) with additional amygdalohippocampectomy achieved favorable seizure outcome compared to 12 of 20 patients (60%) without additional amygdalohippocampectomy (p = 0.06).

### Influence of extent of resection on seizure outcome

The groups of ATL and temporal GTR did not significantly differ in regard of age, sex, tumor localization and MGMT promoter methylation status (Table 3).

**Table 3:** Comparison of ATL and temporal GTR as differing oncosurgical resection modalities

	ATL	Temporal GTR	p value
No. of patients	13 (39)	20 (61)	
Mean age (± SD) (in yrs)	62 ± 13	55 ± 16	0.17
Female sex	5 (38)	9 (45)	1.0
Tumor location			
Dominant hemisphere	8 (62)	11 (55)	1.0
Unmethylated MGMT promotor status	9 (69)	13 (65)	0.7

*ATL* anterior temporal lobectomy, *GTR* gross total resection, *ILAE* International League Against Epilepsy*, MGMT O-6-methylguanine-DNA methyltransferase, SD standard deviation, yrs* years

Values are presented as the number of patients (%) unless stated otherwise

20 patients (61%) with temporal glioblastoma and preoperative TRE underwent GTR, whilst ATL was performed in 13 patients (39%). Patients who underwent ATL for temporal glioblastoma achieved significantly more often favorable seizure outcome (ILAE 1) compared to patients with temporal GTR (13 vs. 10; p = 0.002; OR 27; 95% CI 1.4–515.9).

## Discussion

The concept of supra-total resection strategies extending considerably beyond tumor-enhancing MRI lesions is gaining a growing evidence base in neurosurgical oncology for the surgical treatment of malignant primary brain tumors. In addition to improved overall survival and prolonged progression-free survival interval in previous studies, the present case study also indicates an advantage of a supra-marginal resection strategy with regard to seizure outcome in temporally localized glioblastoma associated with tumor-related epilepsy.

In brain tumors, epilepsy often represents the first symptom and is considered to be a favorable prognostic factor with regard to survival in both low and high-grade gliomas [14–16]. However, TRE leads to a reduced quality of life and can additionally aggravate the burden of living with a brain tumor [17]. To ensure adequate symptomatic therapy of TRE throughout the treatment of these patients, several factors must be considered at different stages. Since tumor related epilepsy is usually based on a local focus, levetiracetam, carbamazepine or phenytoin are often used in the antiepileptic treatment of tumor-related seizures. In some studies, the use of valproic acid (VPA) has often been associated with better overall survival [18–22]. In the context of preoperative treatment of tumor seizures in particular, VPA can be avoided due to the possibility of inducing or aggravating thrombocytopenia.

Nevertheless, in addition to pharmacological therapy, surgical treatment—as evidenced by epilepsy surgery and experiences with low-grade glioma surgery—can also make a decisive contribution to seizure outcome [10, 23, 24]. In a preceding study, we were able to illustrate that a supra-marginal resection strategy in temporal glioblastoma may lead to a significantly prolonged overall survival. In addition to this merely oncological aspect concerning overall survival, we therefore have investigated whether the same procedure might also contribute to seizure outcome in patients with temporal glioblastoma and TRE. Postoperative favorable seizure outcome after initial resection of glioblastomas was previously reported with up to 77% of patients [14]. The results of the present study describing a postoperative seizures freedom in 70% of patients with temporal glioblastomas support these findings.

Anterior temporal lobectomy has already been established in epilepsy surgical research as a useful surgical strategy for alleviating seizures in patients with an epileptogenic focus in the temporal lobe area [25, 26]. In the present study, the performance of ATL for treatment of temporal glioblastoma with TRE was observed to have a significant impact on a favorable seizure outcome in impaired patients (p = 0.002).

Infiltration of the temporo-mesial structures seems to make a significant contribution to the postoperative seizure outcome. In this context, an additional amygdalohippocampectomy was performed in patients with preoperatively proven contrast agent enriching lesions in the area of the temporo-mesial structures. In patients who had a FLAIR component without any contrast enhancement in the temporo-mesial structures, the removal of these structures was at the discretion of the surgeon and was usually not carried out for epileptological considerations, but rather for neuro-oncological considerations regarding a reduction of the local mass in view of an imminent further therapy such as radiation with the potential danger of subsequent reactive tissue swelling. In the literature, especially in the case of low-grade gliomas with accompanying epilepsy, surgical resection of the temporo-mesial structures is associated with an improved seizure outcome [10, 27]. Besides, supramarginal resection might be accompanied by elevated levels of postoperative complications with regard to potentially increased vasculature and eloquent areas at risk which in turn might lead to impaired patients quality of life and worsened OS. In accordance to our previous study on the complication rate in temporal glioblastoma surgery dependent on the extent of temporal glioblastoma resection [12], the present findings do not provide any indication for elevated levels of unfavorable postoperative events in the lobectomy group. This might be reasoned in a reduced risk for postoperative hemorrhage from partially-resected glioblastoma remnants or a potentially reduced occurrence of postoperative increasing reactive peritumoral edema [12, 28, 29]. Nevertheless, surgical interventions in the area of the temporo-mesial structures invariably carry the risk of postoperative neuropsychological impairment. In addition to the tumor burden itself, patients have to deal with eventual side effects of adjuvant therapy and the recurrence therapy that may be necessary during the course of the treatment. Extensive neuropsychological examinations are often not planned in the preoperative evaluation of patients with glioblastomas and often these examinations cannot be undertaken by the patients themselves to the extent practiced in epilepsy/low-grade-glioma surgery due to the often existing tumor-related cognitive limitations. Nevertheless, this aspect is an intriguing perspective for future studies on the neuropsychological assessment of glioblastoma patients, which are intended to undergo supra-marginal resection [30]. The present study endorses the ATL in addition to its known benefit regarding overall survival as the method of choice for surgical control of potential tumor-related seizures in patients with temporal glioblastoma.

### Limitations

The present study has several limitations. The main limitations of this study are the small sample size and the retrospective study design. The surgical procedure of ATL as a paradigm for supratotal resection regimes entails a highly selected cohort of glioblastoma patients with a tumor manifestation within 4 to 5 cm from the temporal pole on the dominant hemisphere and 5 to 6 cm on the non dominant hemisphere. Therefore, a greater sample size is instantly needed in order to increase the validity of our findings. For this purpose, rather multicenter prospective studies will be capable to cope with the restriction of a low incidence of precisely temporal located glioblastoma and thus might create a safe prerequisite to sufficiently assess ATL as a supramarginal resection strategy in temporal glioblastoma disease. Furthermore, estimating the potential effects of radiation and chemotherapy on seizure outcome is complex. However, there are only a few studies assessing the outcome of seizures in patients undergoing glioblastoma resection. The vast majority of studies on glioblastoma resection focus on factors that influence survival or recurrence. Seizure control represents an aspect that has not yet been studied in depth, and has so far been limited to patients with low-grade gliomas. Therefore, the homogeneity of patient/tumor characteristics, methods and results provide an encouraging clinical background for future studies focusing on the influence of the extent of resection on the control of preoperative tumor-related epilepsy in patients suffering from glioblastoma.

## Conclusions

The present study indicates that anterior temporal lobectomy might be associated with a more favorable seizure outcome after temporal glioblastoma resection in terms of a supra-marginal resection strategy compared to GTR. In addition to the described survival advantage, ATL for surgical treatment of temporally localized glioblastoma henceforth seems to be the therapy of first choice given the potential increased seizure freedom.
